# Production and purification of molecular ^225^Ac at CERN-ISOLDE

**DOI:** 10.1007/s10967-024-09811-0

**Published:** 2024-11-15

**Authors:** M. Au, L. Nies, S. Stegemann, M. Athanasakis-Kaklamanakis, T. E. Cocolios, P. Fischer, P. F. Giesel, J. D. Johnson, U. Köster, D. Lange, M. Mougeot, J. Reilly, M. Schlaich, Ch. Schweiger, L. Schweikhard, F. Wienholtz, W. Wojtaczka, Ch. E. Düllmann, S. Rothe

**Affiliations:** 1 CERN, Meyrin, 1211 Geneva Switzerland; 2 Johannes Gutenberg University Mainz, 55099 Mainz, Germany; 3 University of Greifswald, 17489 Greifswald, Germany; 4 KU Leuven, 3001 Leuven, Belgium; 5 Institut Laue-Langevin, 38000 Grenoble, France; 6 Max Planck Institute for Nuclear Physics, 69221 Heidelberg, Germany; 7 University of Manchester, Manchester, M13 9PL UK; 8 Technical University of Darmstadt, 64289 Darmstadt, Germany; 9 GSI Helmholtzzentrum für Schwerionenforschung, 64291 Darmstadt, Germany; 10 Helmholtz Institute Mainz, 55099 Mainz, Germany; 11 Present Address: Centre for Cold Matter, Imperial College London, SW7 2AZ London, United Kingdom; 12 Present Address: Univeristy of Jyväskylä, 40014 Jyväskylä, Finland

**Keywords:** Actinium, Radioactive molecules, Isotope Separation On-Line, Decay spectrometry, Multi-Reflection Time-of-Flight Mass Spectrometry

## Abstract

The radioactive nuclide ^225^Ac is one of the few promising candidates for cancer treatment by targeted-$\alpha$-therapy, but worldwide production of ^225^Ac faces significant limitations. In this work, the Isotope Separation On-Line method was used to produce actinium by irradiating targets made of uranium carbide and thorium carbide with 1.4-GeV protons. Actinium fluoride molecules were formed, ionized through electron impact, then extracted and mass-separated as a beam of molecular ions. The composition of the mass-selected ion beam was verified using time-of-flight mass spectrometry, $\alpha$- and $\gamma$-ray decay spectrometry. Extracted quantities of $^{225}\textrm{Ac}^{19}\textrm{F}_2^{+}$ particles per $\upmu$C of incident protons were $3.9(3)\times 10^7$ from a uranium carbide target and $4.3(4)\times 10^7$ for a thorium carbide target. Using a magnetic mass separator, the long-lived contamination ^227^ Ac is suppressed to $<5.47\times 10^{-7}$ (95% confidence interval) with respect to ^225^Ac by activity. Measured rates scale to collections of 108 kBq$\upmu$A$^{-1}$h$^{-1}$ of directly produced $^{225}\textrm{Ac}^{19}\textrm{F}_2^{+}$.

## Introduction

**Fig. 1:**
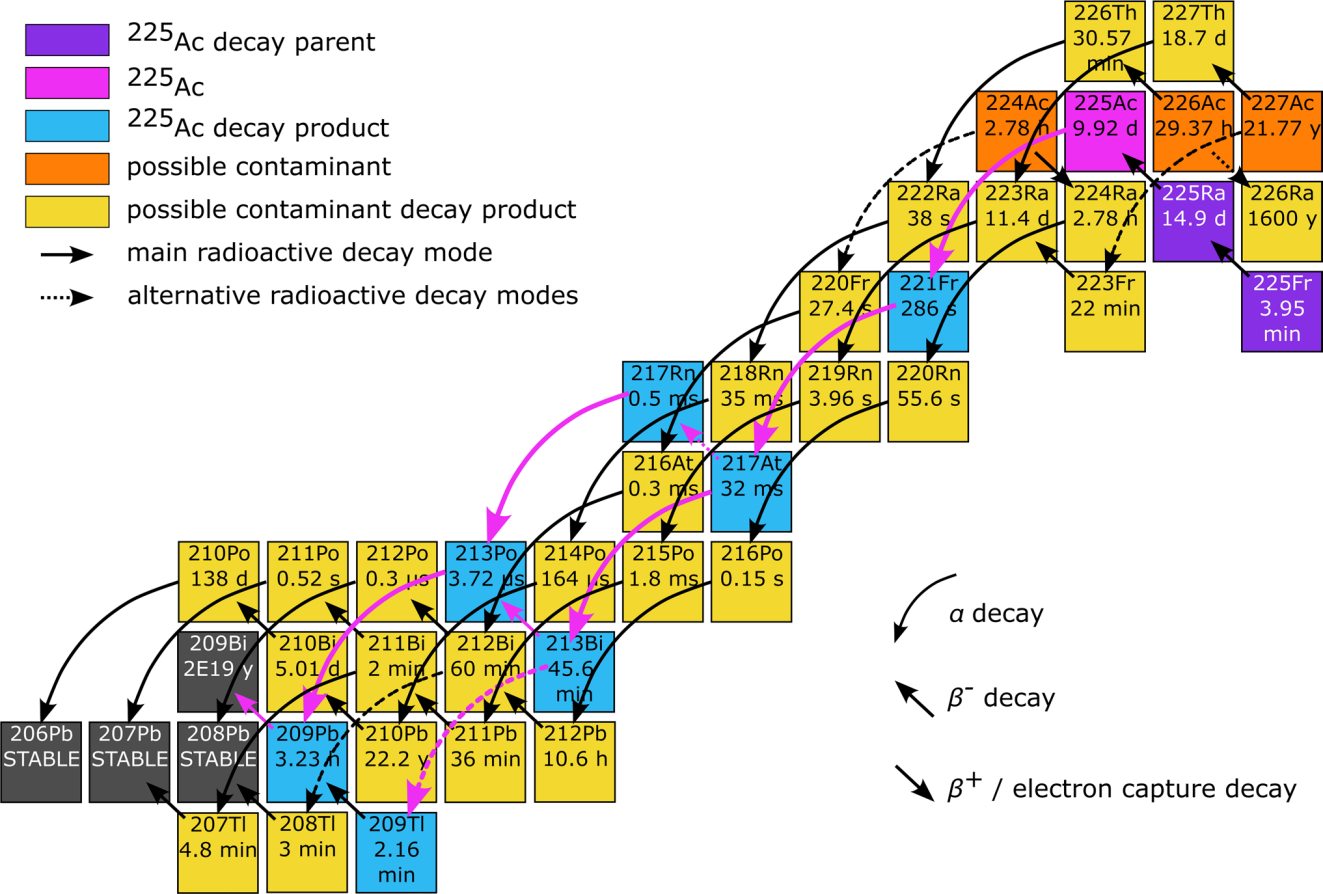
Diagram of the radioactive decay chains of ^225^Ac and co-produced contaminants ^224,226,227^ Ac

In cancer treatment by targeted-$\alpha$-therapy (TAT), tumour cells are damaged by radionuclides decaying through emission of $\alpha$ particles [1]. With a half-life ($t_{1/2}$) of 9.92 days and a decay chain that emits four $\alpha$ particles (shown in Fig. 1), ^225^Ac is one of the few promising candidate radionuclides for TAT [2]. Clinical trials [3, 4] have shown extremely promising responses to TAT treatment, even for prolific and invasive forms of cancer such as gliomas [5]. Despite their therapeutic potential, applications for ^225^Ac are severely limited by the quantity of ^225^Ac available worldwide. Current production routes for ^225^Ac include $\alpha$ decay of its precursor, ^229^Th [6, 7], dominantly available from ^233^U in nuclear legacy material, and accelerator-based approaches including reactions such as ^232^Th(*p*, *x*)^225^Ac [8–10], ^226^Ra(*p*, 2*n*)^225^Ac [11], and ^226^Ra($\gamma ,n$)^225^Ra$\rightarrow ^{225}$Ac [12], usually followed by chemical separation. While production methods based on ^226^Ra or $\alpha$-decay of ^229^Th do not result in co-produced contamination, the accelerator-based methods from Th or U have reported activity fractions of the chemically inseparable and long-lived contaminant ^227^Ac ($t_{1/2}=21.772$ y) ranging from 0.1% [9, 13] to 1% [1] and can add a limitation for safety and handling, clinical studies, and waste management. Recent evaluations [1, 14–18] further discuss existing and considered alternative mechanisms of ^225^Ac production. Improvements in the efficiency, purity, and flexibility of ^225^Ac production processes will facilitate research, development and delivery of new drugs for cancer treatment.

Accelerator-based techniques give opportunities for the production of many radioactive nuclides [19] including ^225^Ac, which can be produced through accelerator-driven nuclear reactions and extracted as a beam of ions at radioactive ion beam facilities [20–22]. The Isotope Separation On-Line (ISOL) method uses nuclear reactions in thick target materials to generate radioactive isotopes. The irradiated targets are typically operated at temperatures above 2100$^{\circ }\text {C}$ to facilitate diffusion of isotopes through and effusion from the target [23, 24] and into the ion source. By employing different ion sources, techniques including ionization by contact with hot surfaces (surface-ionization), resonance laser ionization, and electron-impact and plasma ionization are then used to ionize the isotopes and extract them as a beam of ions [25]. Since both co-produced elements radium and francium are relatively volatile at these temperatures and have low ionization potentials, they are easily extracted by this method. In contrast, actinium is not volatile at these temperatures and is not efficiently surface-ionized, which limits its direct extraction. Release times are on the order of seconds for francium and tens of seconds for radium [26], while actinium shows a lower release efficiency for isotopes with half-lives of less than several days [22]. While the release time should not impact the eventual release of ^225^Ac ($t_{1/2}=$ 9.92 days), the target takes consequently longer to reach equilibrium and can impact scheduling and operation. The use of tunable lasers for element-selective resonance ionization of actinium has been investigated [21, 27]. Laser-ionized ^225^Ac can be extracted and collected along with the surface-ionized parent ^225^Ra [14] which can be used as a generator for ^225^Ac but requires additional radiochemical separation.

Molecular extraction has been used as a technique to extract non-volatile elements from thick ISOL targets via the formation of volatile molecules such as fluorides [28, 29]. In general, the fluorides of actinide elements show large enthalpies of formation suggesting thermal stability [30–32], but many properties of the actinium compounds are still unknown [33, 34]. All isotopes of actinium are radioactive, and only three of these exhibit half-lives longer than one day. The radioactivity and lack of availability pose significant limitations on bulk chemistry studies towards fundamental understanding of actinium complexes, let alone their application in radiopharmaceuticals. This work presents the use of molecular formation to facilitate the extraction of actinium as actinium fluoride molecules at the CERN-ISOLDE facility (1.4-GeV protons, up to 2 $\upmu$A) [35].

Separating the different ion species by their mass-to-charge ratios (*A*/*q*, where *A* is the nucleon number or the sum of nucleon numbers for the molecular constituents, and *q* is the ionic charge state) enables ^225^Ac to be isolated from the heavier ^227^Ac contaminant. Isobaric contaminants for the atomic ion beam, i.e., with $A/q=225$, could include ^225^Fr ($t_{1/2}=3.9$ minutes) and ^225^Ra ($t_{1/2}=14.9$ days), which decay into ^225^Ac by emission of $\beta$ particles (see Fig. 1). In this work the isobaric contaminants and contamination suppression are also evaluated for the use of molecular ion beams $^{225}\textrm{AcF}^+$ ($A/q=244$) and $^{225}\textrm{AcF}_2^+$ ($A/q=263$) where F indicates ^19^F. To the authors’ best knowledge, there are no previous experimental data available in literature on the chemical properties of the ions $\textrm{AcF}^+$ and $\textrm{AcF}_2^+$ produced, identified and applied in this work.

## Methods

**Fig. 2:**
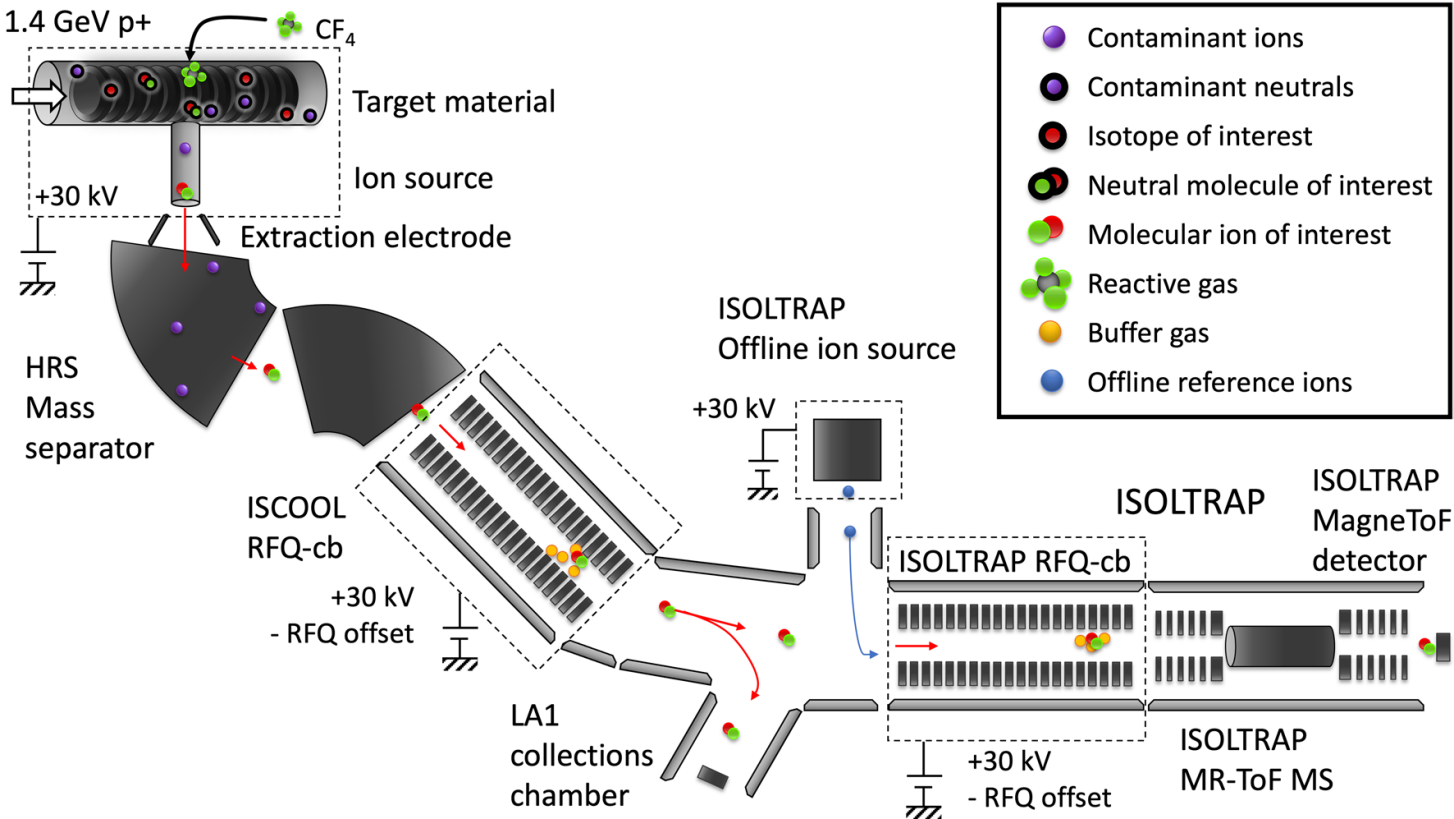
Experimental schematic of radioactive ion beam production and analysis. From upper left: isotopes are generated in the target material with up to 2 $\upmu$A of 1.4-GeV protons from the CERN Proton Synchrotron Booster. Ions are extracted into a beam at 30 kV, separated by their mass-to-charge ratio in the mass separator magnets, then transmitted through the gas-filled ISOLDE RFQ-cooler buncher (ISCOOL). The cooled beams are sent to a collections chamber or to the ISOLTRAP beamline, where the ions are cooled and bunched in the ISOLTRAP RFQ-cooler buncher and sent to the MR-ToF MS

1.4-GeV protons were used to generate nuclear reactions with microstructured porous uranium carbide ($\textrm{UC}_\text {x}$) and thorium carbide ($\textrm{ThC}_\text {x}$) targets at the CERN-ISOLDE facility [35]. Once ^225^Ac is created as a reaction product, it must diffuse out of the target material and effuse to the ion source where it is ionized and extracted as an ion (Fig. 2).

### Molecular formation and ionization

The target units were filled with $\textrm{UC}_\text {x}$ and $\textrm{ThC}_\text {x}$ pills with a total mass of 103.5 g and 95.7 g, respectively. The units were equipped with gas leaks of $1.50(18)\times 10^{-4}$ and $1.30(16)\times 10^{-4}$ mbar $\textrm{L}^{-1}\textrm{s}^{-1}$, respectively, calibrated for helium. The gas mix of argon (Ar) and carbon tetrafluoride ($\textrm{CF}_4$) was applied to the leaks with a pressure of 500 mbar and injected into the transfer line between the target and ion source, providing the fluorine for molecular formation and enabling investigations with different gas compositions. A noble gas mix (He, Ne, Ar, Kr, Xe at 20% each) was used for setup and tuning. The target was resistively heated to temperatures in the range of 1900$^{\circ }\text {C}$ to 2200$^{\circ }\text {C}$, facilitating diffusion of the species of interest from their point of creation in the target matrix to the ion source. The Forced Electron Beam Induced Arc Discharge (FEBIAD) [36] ion source geometry features a tantalum cathode which is heated, typically to temperatures in the range of 2000$^{\circ }\text {C}$ to emit electrons, separated electrically from a molybdenum grid. Electrons from the cathode are accelerated into the anode volume by the anode potential—typically 100-200 V—and confined within a magnetic field, inducing a combination of electron bombardment, plasma ionization, and surface ionization [25]. The ionization technique is not selective and can produce overwhelming contamination in some regions of the nuclear chart. Studying ion beams of the singly-charged actinide fluoride species allows bypassing spallation, fragmentation and fission contamination by operating the mass separator with *A*/*q* larger than that of the target nucleus.

### Ion beam analysis

After ionization, the ions were extracted at 30 kV to ground potential and mass separated by *A*/*q* in ISOLDE’s High-Resolution Separator (HRS), which features two magnetic dipole mass separator magnets in series [35]. The mass-resolving power of the mass separator magnet during the experiment was measured to be $R=m / \Delta m\approx 500$. The mass-separated beam was cooled and transmitted through the ISOLDE radio-frequency quadrupole cooler-buncher (RFQ-cb) [35]. The cooled beams were then sent for further analysis to either of two installations: the ISOLTRAP RFQ-cooler buncher [37] and Multi-Reflection Time-of-Flight Mass Spectrometer (MR-ToF MS) [38] or a vacuum chamber for sample collections by ion beam implantation into aluminum foils. The experimental setup is shown schematically in Fig. 2.

**Fig. 3:**
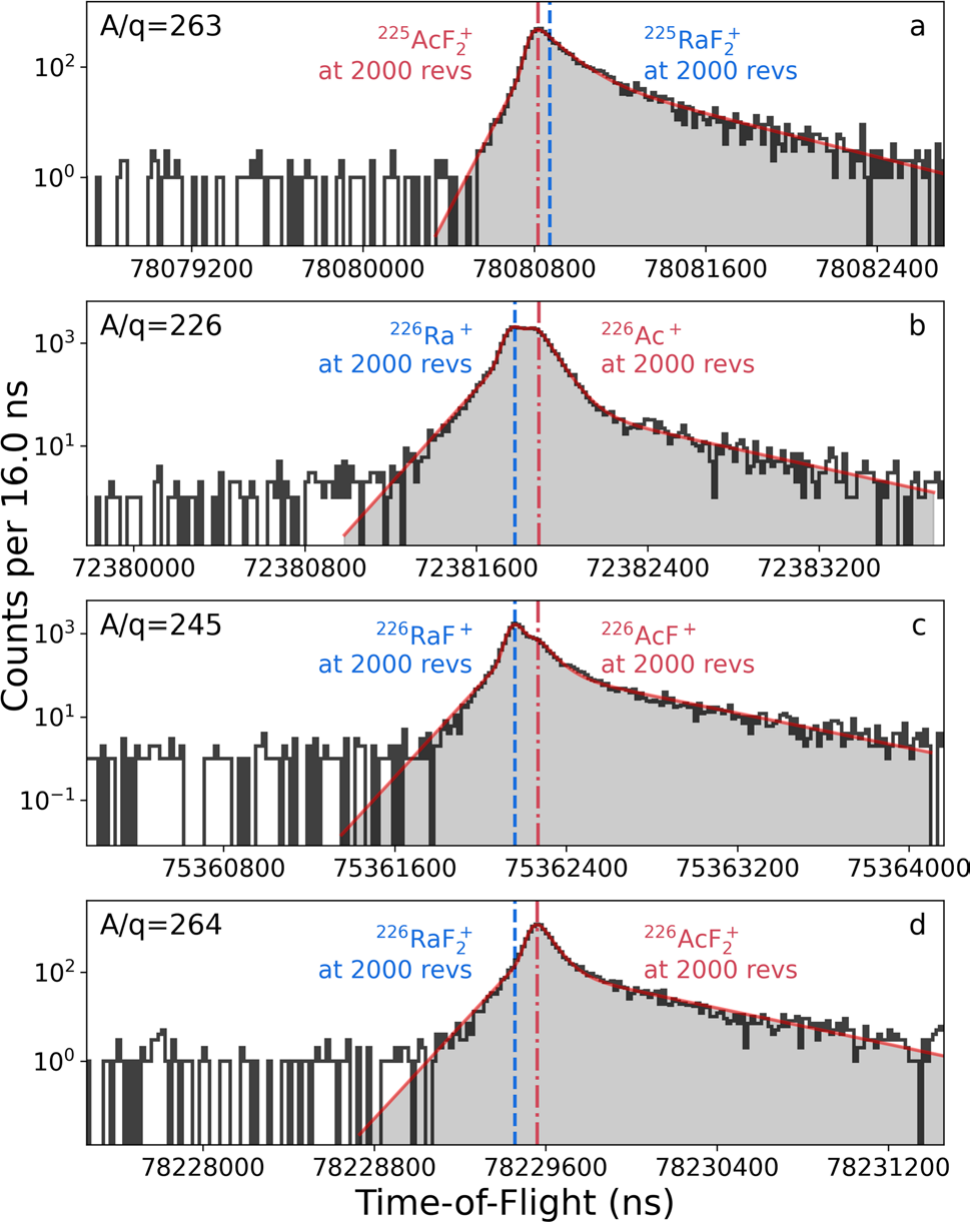
ToF spectra of mass-separated beams from a $\textrm{UC}_{\text {x}}$ target with nominal $A/q=$ **a** 263, **b** 226, **c** 245, and **d** 264 at 2000 revolutions in the MR-ToF MS. Expected ToFs from the calibration are indicated with red (dash-dotted) and blue (dashed) vertical lines for the actinium and radium isobaric species, respectively. The fits to the datasets with the hyper-EMG function [39] are shown as solid red lines

For high-resolution analysis of the ion beam composition using mass spectrometry, the mass-separated beam from HRS was transmitted through the ISOLDE RFQ-cb without storage time. The continuous ion beam was cooled a second time, accumulated, and bunched in the ISOLTRAP RFQ-cb using helium buffer gas. The ion bunch was then injected into the MR-ToF MS and trapped between the electrostatic mirror potentials for typically 1000 revolutions, leading to effective flight paths on the order of a kilometer. The mass-dependent difference in velocity allows ion-bunch components to be separated in time *t* with $R=\frac{t}{2\Delta t}$. Ion arrival times were measured from the RFQ-cb ejection to impact on a MagneToF detector [40] and recorded with 0.8 ns resolution. The MR-ToF MS was calibrated using ^85,87^ Rb and ^133^ Cs from the ISOLTRAP offline ion source and ^238^U from the HRS target and ion source. The asymmetric ToF distributions were fitted using the hyper-Exponentially Modified Gaussian (hyper-EMG) probability distribution function [39] as shown in Fig. 3. By comparison with the ToF calibration, isobaric contaminants in the ion beam were identified. The ISOLTRAP MR-ToF MS routinely achieves mass resolving powers in excess of $10^5$ [41] and has been used to study isobaric and molecular compositions of radioactive ion beams at ISOLDE [38, 42, 43].

Studies by precision mass measurements using the ISOLTRAP MR-ToF MS confirm the identification and support the quantification of the actinium fluorides ($^{225}\textrm{AcF}_2^+$ in Fig. 3a), while also providing information about ion beam purity. Ratios of the molecular sidebands were assessed using the neighboring isotope ^226^Ac ($^{225}t_{1/2}=29$ h) for two reasons: firstly, due to the high mass-resolving power required to separate ^225^Ac from ^225^Ra ($R=590\times 10^3$), ratios of $^{225}\textrm{Ac}^+$ to $^{225}\textrm{Ra}^+$ and $^{225}\textrm{AcF}^+$ to $^{225}\textrm{RaF}^+$ could not be obtained from a ToF spectrum, and it was difficult to conclusively rule out all presence of $^{225}\textrm{RaF}_2^+$ (Fig. 3a). In contrast, separating ^226^Ac from ^226^Ra on the atomic, mono- and di-fluoride species was challenging but possible ($R=330\times 10^3$) as shown in Fig. 3b–d. Secondly, ^225^Ra does not reach secular equilibrium with the decay product ^225^Ac in the target within the duration of the experiment, which could change the ratios of actinium and radium on the time scale of the experimental campaign. For the neighboring isotope ^226^Ac, the long half-life of ^226^Ra prevents the ratio Ac:Ra from changing significantly throughout the duration of the experiment. Therefore, ion beams of $^{226}\textrm{Ac}^+$, $^{226}\textrm{AcF}^+$, and $^{226}\textrm{AcF}_2^+$ were used to evaluate the ratio of actinium nuclides present in each form. CERN-FLUKA [44, 45] calculations modeling 1.4-GeV protons impacting uranium carbide and thorium carbide targets [26] were used to compare the expected in-target production, resulting in rates of 7.71, 7.66, 5.31, and 5.24 (63.8, 79.5, 69.5, and 86.4) for $\textrm{UC}_\text {x}$ ($\textrm{ThC}_\text {x}$) for ^224,225,226,227^Ac, respectively, in units of $10^8$ particles produced per $\upmu$C of 1.4-GeV protons. The ratio of ^225^Ac to ^225^Ra produced is 8, while the ratio of ^226^Ac to ^226^Ra is 6.3. Molecular formation is expected to depend on electronic configuration and the conditions in the hot cathode FEBIAD-type ion source which, in particular, can lead to fragmentation. For isotopes of the same element, the effect of different neutron numbers on the electronic configuration (the isotope effect) is expected to have practically no influence on the ratio of observed molecules.

### Sample collection and measurement

**Fig. 4:**
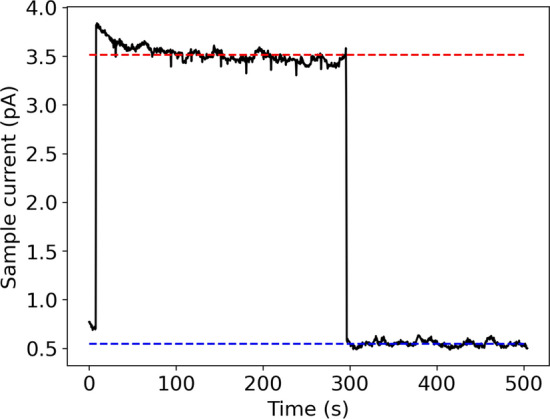
Measured ion current on the sample holder during a 288 s-implantation of $^{225}\textrm{AcF}^+_2$ from a $\textrm{UC}_\text {x}$ target at 2050$^{\circ }\text {C}$. The average ion current during implantation is indicated with a red dashed line and the average background reading without incident ion beam is indicated in blue. Values shown are after correction for secondary electron emission

Complementary to ToF identification (Fig. 3), ion implantations followed by decay-spectrometry measurements were used to quantify ^225^Ac and its parent ^225^Ra present as atomic ion beams and molecular sidebands. Samples of the mass-separated ion beam were implanted into aluminum collection foils positioned on a six-sided sample holder in the LA1 beamline (indicated by LA1 collections chamber in Fig. 2). The sample holder ion current was recorded during each collection (e.g., Fig. 4) in addition to the corresponding ion current on an upstream collimator and the number of protons received by the target. No bias voltage for secondary electron suppression was applied to the sample holder. The detected current was corrected to the equivalent ion beam intensity by calibration with a Faraday cup (secondary electron suppression voltage 60 V). The transport efficiency measured using Faraday cup ion current readings after mass separation and before the implantation setup was approximately 40%. The most significant losses occurred in the gas-filled ISCOOL RFQ-cb, which is a component of the ISOLDE HRS mass separator used for this experiment. An RFQ-cb is not required for producing molecular ion beams.

The samples were taken to an off-line $\alpha$-decay spectrometer (ORTEC Alpha Aria [46]) and measured using a Canberra passivated implanted planar silicon (PIPS) detector. For both the $\textrm{UC}_\text {x}$ and $\textrm{ThC}_\text {x}$ experiments, the $\alpha$-particle detector was calibrated (energy, efficiency) in the days prior to starting the measurements.

**Fig. 5:**
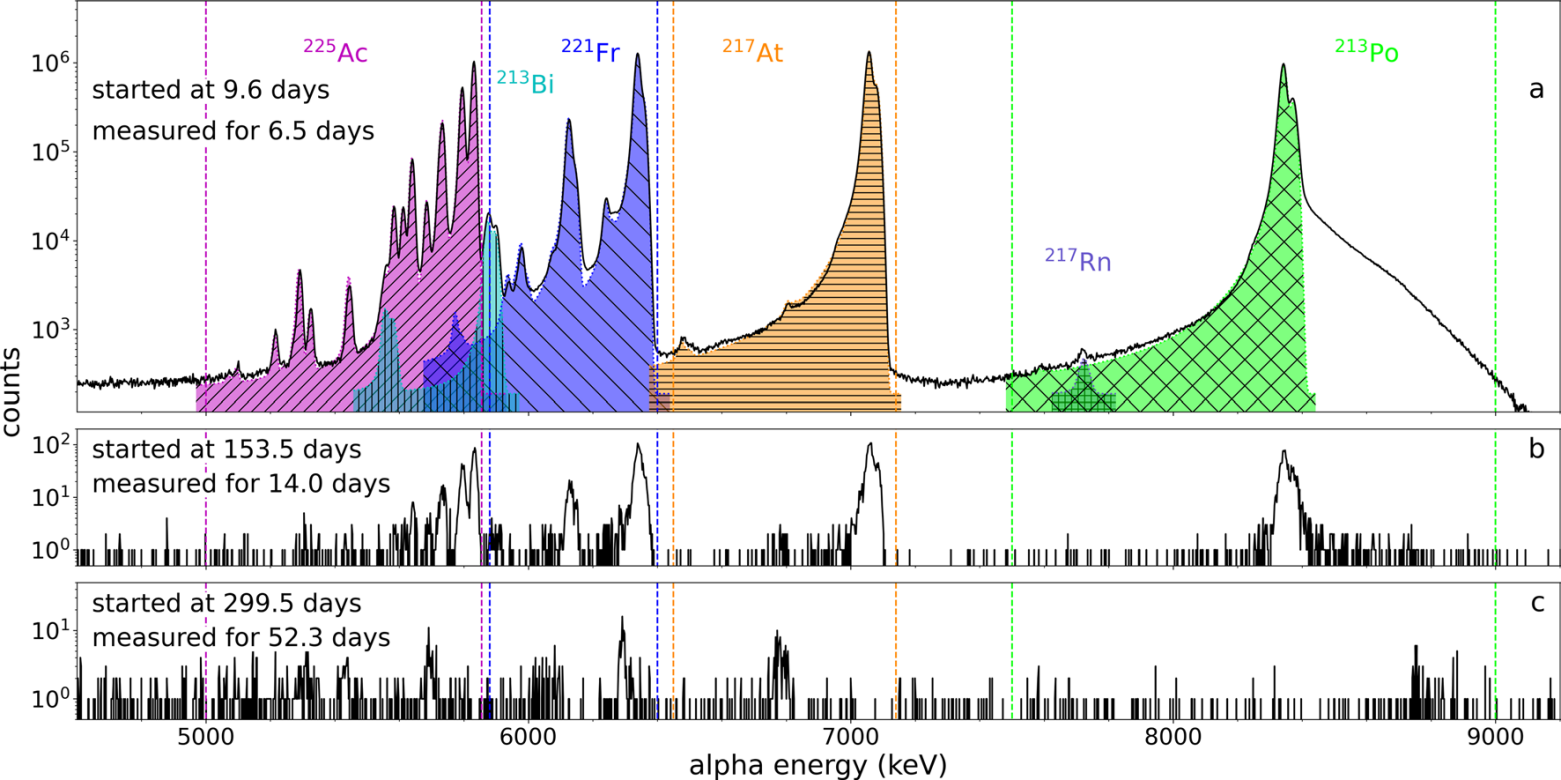
$\alpha$ decay spectrum of an ion-implanted sample collected on $A/q=263$ ($^{225}\textrm{AcF}_2^+$) from a $\textrm{UC}_\text {x}$ target at 2067(50)$^{\circ }\text {C}$ with a gas mix of 40% $\textrm{CF}_4$, 60% Ar (see also Table 1). The spectra start times and measurement lengths are indicated on the left. Characteristic $\alpha$ decays of ^225^Ac, and its decay products ^221^Fr, ^217^At, ^217^Rn, ^213^Po and ^213^Bi, are described by a sum model of Crystal Ball functions [47] and shown in shaded colours (magenta, dark blue, orange, purple, green, and light blue, respectively) in panel a. Dashed vertical lines show regions used for counting

Figure 5 shows an $\alpha$-decay spectrum for the ^225^Ac nucleus and its decay products. The ranges used for counting $\alpha$ decays of each species are indicated with dashed lines. The observed spectrum is described by an analytical function for peaks in $\alpha$-particle spectra from Si detectors presented by the Crystal Ball collaboration [47]. The $\alpha$-decay spectrum from an ion-implanted sample collected on $A/q=263$ (^225^$\textrm{AcF}_2^+$) displays $\alpha$-decay energies and heights corresponding to the branching ratios available in literature [48–51], for the characteristic decay of the ^225^Ac nuclear ground state. Further details on the analysis of the $\alpha$-decay spectrum are given in Appendix 5.

The samples were also measured by $\gamma$-ray spectrometry using a high-purity germanium HPGe detector. Eight $\gamma$-ray lines corresponding to ^225^Ac were observed, in addition to two lines of the daughter ^221^Fr and lines from ^213^Bi and ^209^ Th which are also nuclides in the ^225^Ac decay chain. The spectrum further supports the identification concluded from the $\alpha$-decay spectrometry and the ToF mass measurements. Further details can be found in Appendix 5.

**Fig. 6:**
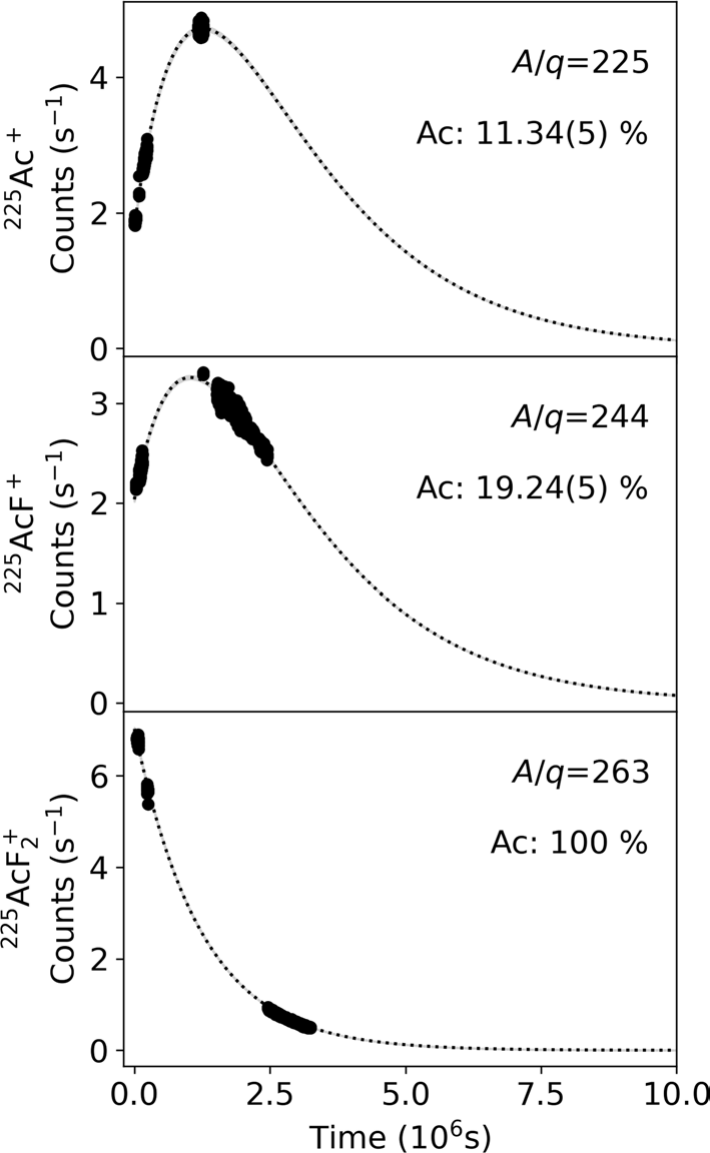
Measured counts per second of $\alpha$-decays for ^225^Ac from samples collected as atomic, monofluoride, and difluoride ion beams. Samples were collected for 43.2 s each from a $\textrm{ThC}_\text {x}$ target at 2050$^{\circ }\text {C}$ with $4.6\times 10^{14}$ particles per second of 40% $\textrm{CF}_4$ and 60% Ar with the mass separator magnet set to $A/q=$ 225, 244 and 263. Counts shown are before correction for geometrical detection efficiency and branching ratios. Fits describing the activity of ^225^Ac originating from initial amounts of ^225^Ac and its parent ^225^Ra are shown with dotted lines. Variation of the fitted parameters within 3$\sigma$ leads to values within the thickness of the dotted line (Further details in section 2.3). The initial fraction of actinium ions with respect to the total number of isobaric ions (Fr, Ra, Ac), extracted from the fit, is indicated in %

Samples of the ion beam at mass setting $A/q=$225, 244, and 263 were collected for times ranging from 38.4 s to 288 s and measured as demonstrated in Fig. 6. The yield of ^225^Ac nuclei from ion beams on each mass setting was calculated per number of protons incident on the target by measuring the number of actinium nuclei collected during a given accumulation time and corrected for transmission. The $\alpha$ decays detected in the energy range for ^225^Ac were recorded for the three samples after the ion-beam collection. The samples show a grow-in of ^225^Ac from the decay of the parent ^225^Ra for the atomic ($A/q=225$) and monofluoride ($A/q=244$) species. ^225^Fr (half-life 3.95 min) decayed into ^225^Ra during the time required to transport the samples to the detector, which was at minimum four ^225^Fr half-lives. The possible contribution of ^225^Fr to the atomic sample was thus not observed.

### Contaminant evaluation

Decay spectrometry was used to evaluate an upper limit for the suppression of neighboring masses, to $A/q\pm 2$, achieved by the mass-separator dipole magnets when collecting $^{225}\textrm{AcF}_2^+$, particularly for long-lived contaminants such as ^227^Ac. The mass separator is expected to suppress the two neighboring isotopes ^224,226^Ac by approximately equal amounts, and ^227^Ac is expected to be suppressed by significantly more. The sample of $^{225}\textrm{AcF}_2^+$ used for the limit was collected for 288 s from a $\textrm{UC}_\text {x}$ target at 2067(50) $^{\circ }\text {C}$ with a gas flow rate of $5.1(2)\times 10^{14}$ particles per second of a gas mix of 40 %$\textrm{CF}_4$, 60 %Ar.

The $\gamma$-ray spectrometry was performed three days after the collection finished and used to derive an upper limit of ^222^Ac to ^225^Ac by number of atoms for the lighter neighboring isotope ^222^Ac ($t_{1/2}=2.78$ h). No $\gamma$-lines were observed for the heavier neighboring isotope ^226^Ac ($t_{1/2}=29.37$ h). The $\alpha$ decay (5304.33 keV with intensity 100 %) of the decay product ^210^Po ($t_{1/2}=138.376$ d) was used to evaluate a limit of ^226^Ac atoms in the sample.

The $\alpha$-decay branch of ^227^Ac (4953.26(14) and 4940.7(8) keV with 0.658(14) and 0.546(17) % intensities, respectively [52]) and the decay product ^215^ Po (7386.1(8) keV with an $\alpha$-decay branch of 99.99977(2)% [53]) were used to evaluate a limit for the suppression of ^227^$\textrm{AcF}_2^+$. Since ^225^Ac also has $\alpha$ decays in the range of the ^227^Ac $\alpha$-particle energies, the upper-limit measurement of ^227^Ac contamination was performed 300 days after the end of the collection to allow for the complete decay of ^225^Ac (30.2 half-lives) as shown in Fig. 5. For further details see Appendix 5.

## Results

### Identification and beam purity evaluation

The $\alpha$-decay spectrum of the sample in which the molecular beam ^225^$\textrm{AcF}_2^+$ ($A/q=263$) was implanted, shown in Fig. 5, matches the characteristic $\alpha$-decay spectrum of the ^225^Ac ground state and its decay products using a sum model of 45 peaks and resolving $\alpha$ decays with intensities as low as 0.003 %, unambiguously confirming the presence of ^225^Ac on the mass setting $A/q=263$ for both $\textrm{UC}_\text {x}$ (shown in Fig. 5) and $\textrm{ThC}_\text {x}$ targets. The $\alpha$ particle count rate from $\alpha$-decay spectrometry measurements of the ion-implanted foils shown in Fig. 6 indicates the presence of the parent ^225^Ra for the atomic ($A/q=225$) and monofluoride ($A/q=244$) species, observed by the initial increase of ^225^Ac activity caused by feeding from the decay of ^225^Ra. The rate of ^225^Ac $\alpha$ decays from the difluoride sample ($A/q=263$) shows no identifiable presence of ^225^Ra.

Precision mass measurements are in agreement with the presence of ^225^Ac and its parent ^225^Ra identified through $\alpha$-decay spectrometry. While the $\alpha$-decay spectrometry can identify nuclides that decay by emission of an $\alpha$-particle, precision ToF mass measurements can identify ion beam components regardless of decay mode or half-life. The ToF spectrum (Fig. 3a) confirms the presence of ^225^$\textrm{AcF}_2^+$ and shows no other notable components in the ion beam. The purity and ion beam compositions of $^{226}\textrm{Ac}^+$, $^{226}\textrm{AcF}^+$, and $^{226}\textrm{AcF}_2^+$ were determined by evaluating the fraction of actinium nuclides present in each ToF signal (detailed further in 2.2). The corresponding ToF spectra are shown in Fig. 3b–d. On the atomic mass setting ($A/q=226$), $^{226}\textrm{Ac}^+$ accounted for 41.7(8)% of the ion beam, in addition to $^{226}\textrm{Ra}^+$. On the mono-fluoride mass setting ($A/q=245$), $^{226}\textrm{RaF}^+$ was also more intense than $^{226}\textrm{AcF}^+$, which formed only 15.0(2)% of the ion beam. On the di-fluoride mass ($A/q=264$), the ion beam was purely composed of $^{226}\textrm{AcF}_2^+$ with no detected contamination. On the atomic mass setting, ^226^Fr was present in negligible amounts from the $\textrm{UC}_\text {x}$ target under the conditions of these experiments, while Fr was observed at higher rates from the $^{226}\textrm{ThC}_\text {x}$ target, following the expected in-target production rates which are 10 and 6 times higher from $\textrm{ThC}_\text {x}$ than from $\textrm{UC}_\text {x}$ for ^225^Fr and ^226^Fr, respectively. The decay behavior observed for ion beams collected on the three mass settings in Fig. 6 agrees with the observations made through ToF identification in Fig. 3.

### Quantification of produced ^225^Ac

The signature $\alpha$ decay of ^225^Ac gives the measured number of ^225^Ac nuclei per charge of primary proton (ions per $\upmu$ C) incident on the target during the ion collection as shown in Table 1 for $\textrm{UC}_\text {x}$ and $\textrm{ThC}_\text {x}$ targets. The yield can equivalently be considered as a rate of ^225^Ac species in ions per second per $\upmu$ A of protons. The simulated number of ^225^Ac nuclei produced in the target per 1 $\upmu$C of 1.4-GeV protons is one order of magnitude higher for $\textrm{ThC}_\text {x}$ targets than for $\textrm{UC}_\text {x}$ targets ($7.95\times 10^9$ compared to $7.66\times 10^8$) using the CERN-FLUKA Monte-Carlo code [26, 54]. The ionization efficiency was benchmarked throughout both experiments using the observed ion rates from the calibrated leaks of argon gas and was between 1 and 5 % for the range of ion source temperatures used. Ionization efficiencies were stable within 1 % while maintaining constant conditions. We observe similar yields from the $\textrm{ThC}_\text {x}$ and $\textrm{UC}_\text {x}$ targets studied in this work, resulting in a difference in efficiency corresponding to the difference in simulated in-target production between the two materials. It is important to note that efficiencies reported here compare the rate of ions extracted as ^225^$\textrm{AcF}_2^+$ to the rate of ^225^Ac nuclides produced in the target.

**Table 1:** Yield from $\textrm{UC}_\text {x}$ (67.2 $\textrm{g cm}^{2}$)and $\textrm{ThC}_\text {x}$ (62.2 $\textrm{g cm}^{2}$) targets of $^{225}\textrm{AcF}_2^+$ as collected samples of ions implanted into aluminum foil and measured using $\alpha$-decay spectrometry.

Target Material	$\textrm{CF}_4$:Ar ratio	$\textrm{CF}_4$ rate (nmol $\textrm{s}^{-1}$)	Target temperature ($^\circ$C)	Yield (ions $\mu$ C ^-1^)	Total efficiency (%)
$\textrm{UC}_\text {x}$	10:90	0.065(8)	2028(50)	3.7(5)$\times 10^5$	0.049(6)
$\textrm{UC}_\text {x}$	20:80	0.13(2)	2028(50)	9(1)$\times 10^5$	0.12(2)
$\textrm{UC}_\text {x}$	40:60	0.26(3)	2067(50)	3.9(3)$\times 10^7$	5.1(5)
$\textrm{ThC}_\text {x}$	40:60	0.22(3)	1930(50)	1.3(1)$\times 10^7$	0.16(1)
$\textrm{ThC}_\text {x}$	40:60	0.22(3)	2050(50)	2.0(2)$\times 10^7$	0.26(3)
$\textrm{ThC}_\text {x}$	100: -	0.55(7)	2150(50)	4.3(4)$\times 10^7$	0.54(5)

Forced Electron Beam Induced Arc Discharge (FEBIAD)-type ion sources [36] were used with different gas mixes and target temperatures. Efficiency with experimental uncertainty is given with respect to the in-target production rate of ^225^Ac predicted from CERN-FLUKA Monte-Carlo simulations [44, 45], calculated per $\upmu$ C of 1.4-GeV protons incident on the target material [26]. The target and ion source configuration and operation are described in more detail in Sect. 2

### Upper limit of contamination

The application of ^225^Ac as a radiopharmaceutical imposes stringent limits on acceptable contamination by other nuclides, particularly radionuclides with long half-lives.

The $\gamma$-ray spectrometry performed three days after the collection finished was used to derive an upper limit of $<8.8\times 10^{-6}$ ^222^Ac to ^225^Ac by number of atoms for the lighter neighboring isotope ^222^Ac ($t_{1/2}=2.78$ h). No $\gamma$ lines were observed for the heavier neighboring isotope ^226^Ac ($t_{1/2}=29.37$ h). The $\alpha$ decay (5304.33 keV with intensity 100 %) of the decay product ^210^ Po ($t_{1/2}=138.376$ d) was used to evaluate a limit of $<7.35\times 10^7$^226^Ac atoms in the sample, resulting in a suppression of $<1.6\times 10^{-2}$ by number of atoms.

The spectrum used for the ^227^Ac contaminant analysis was measured for 52.3 days, starting 300 days after the collection (Fig. 5c). Since the $\alpha$-decay branch of ^227^Ac is small and its energy range (4920 to 4975 keV) overlaps with some low intensity $\alpha$-decay lines of ^225^Ac, the $\alpha$ decay of the daughter ^215^ Po (7371 to 7401 keV) was used to set an upper limit for the possible ^227^Ac fraction. The upper limit using the Currie equation [55] gives $<5.47\times 10^{-7}$ at 95 % confidence interval for the ^227^AcF_2_^+^ to ^225^AcF_2_^+^ ratio by activity.

## Discussion

A selection of compounds have been observed with actinium in the form Ac ^3+^, in which it has a noble gas (closed-shell) configuration of 86 electrons [34]. Few experimental data exist for actinium compounds in which actinium takes other oxidation states. The production and identification of $\textrm{AcF}^+$ containing the isotopes ^225,226^Ac gives experimental evidence for the existence of actinium compounds in which actinium may take an oxidation state other than $\textrm{Ac}^{3+}$. The production of $\textrm{AcF}^+$ and $\textrm{AcF}_2^+$ molecules at a radioactive ion beam facility opens the doors for systematic experimental studies on these species towards research on radioactive molecules [56, 57] and specifically the physical chemistry of actinium, for which there is a general lack of data.

The combination of time-of-flight mass measurements and $\alpha$-decay spectrometry shows unambiguously that ^225^Ac is delivered in the form $\textrm{AcF}_2^+$ as a pure beam. The ion beam compositions for the atomic, mono- and di-fluoride species offer different possibilities for collections of ^225^Ac by including or excluding the isobaric components ^225^Ra and potentially ^225^Fr, which are present as atomic ions ($A/q=225$), with the ^225^Ra decaying by emission of a $\beta$ particle into ^225^Ac. Alternatively, a combined beam of $\textrm{AcF}^+$ and $\textrm{RaF}^+$ ($A/q=244$) can be collected without ^225^Fr. Pure samples of ^225^Ac can be obtained in the form of $\textrm{AcF}_2^+$ ($A/q=263$). To the authors’ knowledge, $\textrm{FrF}^+$ and $\textrm{RaF}_2^+$ beams have not been observed at CERN-ISOLDE for any isotope of Fr and Ra, respectively. Simultaneous collection of ion beams on all three *A*/*q* settings would increase the final amount of ^225^Ac collected, but would require a dedicated mass-separation system.

The long-lived and chemically-inseparable contaminant ^227^Ac was suppressed to $<5.47\times 10^{-7}$ ^227^Ac to ^225^Ac by activity, significantly exceeding the purity of directly produced accelerator-based ^225^Ac which is on the order of 0.1 % [1]. This method enables direct production with purity comparable to or better than that of ^225^Ac indirectly produced through ^225^Ra generator systems, reported to be $<7.5\times 10^{-7}$ [13].

The technique is demonstrated in this work for actinium produced in proton-induced spallation reactions in thorium and uranium targets. Appreciable cross-sections of ^225^Ac production occur for proton energies above 100 MeV for thorium [58–60] and hundreds of MeV for uranium targets [16]. The technique of molecular extraction is independent of the irradiation type but, importantly, depends strongly on the material structure and composition of the target. The target material should be carefully considered if scaling the rates found in the present study regarding expected production of ^225^Ac, ^225^Ra, and ^225^Fr for other facilities with different incident particle intensities and energy- and particle-dependent reaction cross-sections.

The target temperature during the experiment did not exceed 2150$^{\circ }\text {C}$, demonstrating that ion beams of actinium fluorides can be extracted at typical operation conditions for actinide carbide ISOL targets using this molecular extraction technique. At comparable temperatures, measured rates of laser-ionized Ac are the same or slightly lower [22]. Yields reported here are conservative—higher yields could likely be observed with operation above nominal target temperatures as discussed in Refs. [14, 23] at the possible cost of increased target ageing through sintering of the open-porous microstructure. The collections performed at the same target temperature (with the same heating current applied to the target, Table 1), show a difference in total efficiency that scales approximately linearly with the percentage of $\textrm{CF}_4$ in the gas mixture. The yield increase observed after the increase of $\textrm{CF}_4$ gas content in the gas mixture suggests that at nominal temperatures, the extraction of actinium was improved by molecular formation. The efficiency reported here is given for collection of ^225^Ac^19^$\textrm{F}_2^+$ only, with respect to the calculated production per charge of incident 1.4-GeV protons ($7.66\times 10^8$ per 1 $\upmu$C [26]). The technique was not tested until target failure in this work. Further systematic optimization of production conditions including temperature and $\textrm{CF}_4$ content could improve the process efficiency.

The yields measured at the tested conditions per 1 $\upmu$C for both the $\textrm{UC}_\text {x}$ and $\textrm{ThC}_\text {x}$ targets translate to approximately 30 Bq collected for one second of collection time for pure ^225^Ac in the form $\textrm{AcF}_2^+$, or 108 kBq of ^225^Ac activity collected per 1 $\upmu$Ah for targets with similar thickness around 65 g per $\textrm{cm}^{2}$. Clinical trials [4] have demonstrated promising results with the administration of 100 kBq per kilogram of patient body weight [61], or dosages on the order of 10 MBq. Recent cross-section measurements predict 7-14 GBq of ^225^Ac produced in 10 days of irradiation at Los Alamos National Lab (250 $\upmu$A protons, up to 100 MeV) and 13.6 GBq at Brookhaven National Lab (130 $\upmu$A protons, up to 200 MeV) [8]. TRIUMF’s Isotope Production Facility (up to 500 MeV) produced 521 MBq of ^225^Ac and 91 MBq of ^225^Ra over total integrated irradiations of 2640 $\upmu$Ah (36–41  days) [13].

The method of molecular extraction and subsequent mass-separation is characterized at CERN-ISOLDE, demonstrating extraction of the produced ^225^Ac as a molecular ion with efficiencies two times higher than the atomic ion at comparable conditions [22] while eliminating the non-isobaric ^227^Ac contaminant and offering the option to collect or exclude the parent nuclide ^225^Ra. At present, this technique could be applied to the production of high-purity samples, with or without the parent nucleus ^225^Ra, for development of new chelating agents and studies on the chemistry of ^225^Ac. Notably, CERN-ISOLDE operates with a maximum driver beam intensity of 2 $\upmu$A of high-energy (1.4 GeV) protons, and unlike the facilities mentioned above, it is not a facility designed or intended for regular high-intensity production of medical isotopes. If the rates measured in this work were to be scaled per $\upmu$ A to the proton intensities available at the high-intensity driver beam facilities built for medical isotope production, the result could be comparable collection rates of GBq of pure ^225^Ac after days of ion collection. This method could allow simultaneous production and collection of ^225^Ac as well as the flexibility to implant the ions onto different desired substrates for further processing, with collection rates of one dose (10 MBq) per 4 $\upmu$Ad. This work demonstrates, characterizes, and quantifies an additional method which could be considered in the global effort to scale up the production of ^225^Ac for cancer treatment by TAT.

## Figures and Tables

**Fig. 7:**
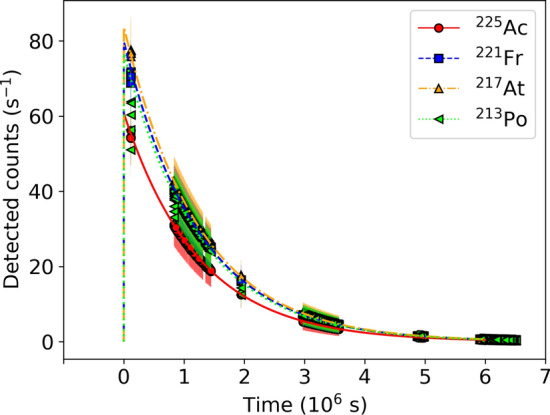
Measured counts per second of $\alpha$ decays for ^225^Ac and daughter nuclei of a sample collected for 288 s from a $\textrm{UC}_\text {x}$ target at 2067$^{\circ }\text {C}$ with the mass separator magnet set to $A/q= 263$. Counts shown are before correction for geometrical detection efficiency and branching ratios. Fits from the corresponding Bateman equation are shown with solid and dotted lines

**Fig. 8:**
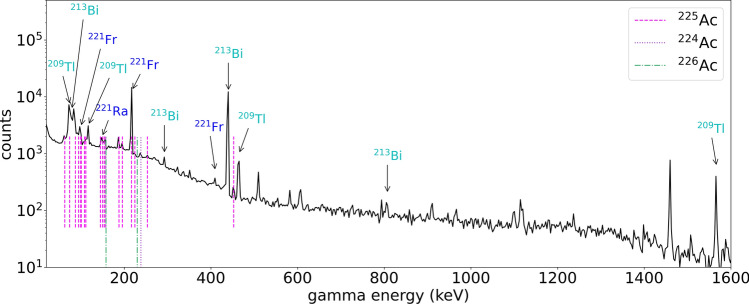
Measured counts of $\gamma$ decays for ^225^Ac and its decay products from of a sample collected for 288 s from a $\textrm{UC}_\text {x}$ target at 2067$^{\circ }\text {C}$ with the mass separator magnet set to $A/q= 263$. Counts shown are before correction for energy or geometrical detection efficiency. Dashed lines indicate known $\gamma$ lines of ^225^Ac, dotted and dash-dotted lines indicate lines for evaluation of possible contaminants ^224,226^Ac

## Decay spectrum analysis

The $\alpha$-particle detector was calibrated (energy, efficiency) using a sample with four $\alpha$-particle emitters: ^148^Gd (1.167 kBq ± 3.24%), ^239^Pu (1.057 kBq ± 3.24%), ^241^ Am (1.186 kBq ± 3.24%), and ^244^Cm (1.190 kBq ± 3.23%), as specified by the manufacturer on November 1, 2007. For both the $\text {UC}_\text {x}$ and $\text {ThC}_\text {x}$ experiments, the calibration was performed in the days prior to starting the measurements. The detection efficiency in the measurement position was evaluated to be 1.6(1) %, with typical peak full-width at half-maximum (FWHM) 16.5(1.7) keV.

In the collected $\alpha$-decay spectrum shown in Fig. 5, satellite-peak structures from the implantation of recoil nuclei in the detector are observed for the daughter nuclei, resulting in a second peak structure for ^221^Fr, ^217^At, ^213^Po and ^213^Bi shifted upwards by the kinetic energy of the recoiling nucleus [62]. The high-energy tailing observed for the ^213^Po peak is due to energy summing of electrons from the $\beta$ decay of the ^213^Bi precursor and $\alpha$ particles emitted by ^213^Po within the shaping time of the amplifier, which is comparable to the 4.2 $\upmu$s half-life of ^213^Po. Recoil nuclei in a decay chain have an additional factor in geometrical efficiency resulting from implantation in the walls of the measurement chamber.

A second model of the spectrum was created using a recently updated dataset in Ref. [63] for comparison, achieving good visual agreement with the spectrum presented in Fig. 5. For the 5321.2-keV $\alpha$ line, the LNHB dataset reports a probability of 0.007 (7) % [63] while the previously published dataset in Nuclear Data Sheets from 2007 reported 0.070 (10) % [50] for this same line. The spectrum in Fig. 5 shows better agreement with the Nuclear Data Sheets probability of 0.07 %.

The Bateman equation [64],

1 $$\begin{aligned} N_n(t) =N_0(0)\times \left(\prod _{i=0}^{n-1}\lambda _i\right)\times \sum _{i=0}^{n}\frac{e^{-\lambda _it}}{\prod _{j=0,j\ne i}^{n}(\lambda _j-\lambda _i)} \end{aligned}$$

where $N_n$ with $n=0,1,2,3$ indicating ^225^Ac, ^221^Fr, ^217^At, and ^213^Po, respectively, is used to describe the populations of the four nuclei in the decay chain. $N_n$ is the number of atoms and $\lambda _i$ is the decay constant $\frac{\ln 2}{t_{1/2}}$ with $i=0,1,2,3$ (Fig. 7). The time $t=0$ is taken as the end of the collection (tens to hundreds of seconds after the start of collection) during which decay of collected ^225^Ac is negligible with respect to the 9.92-day half-life. The measured activity of each isotope using the counting region shown in Fig. 5 was fitted using the corresponding Bateman equation with initial amounts of ^225^Ac and ^225^Ra as the only free parameters. Recoil products follow the expected exponential decay for the decay chain in addition to ingrowth observed for short-lived daughter nuclides after the sample was removed from and then returned to the measurement position. The observed offset in the number of detected counts shown in Fig. 7 is caused by the slightly higher geometrical efficiency for the recoil nuclei, evaluated to be 0.5%, 0.6%, and 0.4% for the first, second, and third decay product respectively in addition to the 1.6(1) % detection efficiency from the sample position. At a beam energy of 30 keV, the ions are expected to be implanted approximately 20 nm below the foil surface [65]. Energy loss for the $\alpha$ decays of ^221^Fr, ^217^At and ^213^Po resulting from the ion implantation depth is expected to be less than 4 keV, resulting in a small shift in observed peak centres with respect to the external calibration $\alpha$ source [66].

The samples were measured by $\gamma$-ray spectrometry using a high-purity germanium HPGe well detector (Canberra) with a digital spectrum analyzer (DSA-1000, Canberra) operated with the GENIE 2000 Gamma Analysis Software Package Nuclide Identification and Quantification [67]. The energy *E* and efficiency of HPGe $\gamma$-ray detection $\epsilon _{\gamma }$ were calibrated using a ^152^Eu sample, giving an energy-dependent efficiency: $\epsilon _{\gamma } = \exp (-161.9+102.7\ln E-24.92\ln E^2 + 2.661\ln E^3 + 0.1063\ln E^4)$ with a typical peak FWHM$=2.6(1)+0.013E$ (all *E* values in keV). Eight lines corresponding to ^225^Ac were observed: an inseparable doublet (99.80 keV with intensity 1.00(11) % and 99.60 keV with intensity 0.70(6) %) with a combined intensity of 1.70(13) %, as well as 150.10 keV with 0.60(3) % intensity. In particular, the lines 62.9 keV with 0.43(2) % intensity, 188.0 keV with 0.45(2) % intensity, 195.8 keV with 0.123(6) % intensity, 253.5 keV with 0.116(6) % intensity, and 452.4 keV with 0.089(5) % intensity, were relatively clean.

Two $\gamma$-ray lines of the daughter ^221^Fr (218.00 keV with 11.44(12) % intensity and 410.40 keV with 0.1205(24) % intensity) were also observed as well as $\gamma$-ray lines from ^213^Bi (440.45 keV with 25.9(2) % intensity and 292.8 keV with 0.419(8) % intensity) and ^209^Tl (218.00 keV with 11.44(12) % intensity and 410.40 keV with 0.1205(24) % intensity). Figure 8 shows these and other possible contributions to the $\gamma$ spectrum from the decay chain of ^225^Ac.

To derive the upper limit for ^222^Ac ($t_{1/2}=2.78$ h), its decay product ^212^Pb (238.63 keV with intensity 43.6(5) %) was used to set an upper limit of $<2.2\times 10^4$ atoms, or a ratio of $<8.8\times 10^{-6}$ ^222^Ac to ^225^Ac by number of atoms. No $\gamma$ rays of the heavier neighboring isotope ^226^Ac ($t_{1/2}=29.37$ h) were observed.
